# HTA on neonatal screening for rare metabolic disorders faced misconceptions and blurred objectivity

**DOI:** 10.1186/1750-1172-7-S2-A17

**Published:** 2012-11-22

**Authors:** Ilona Autti-Rämö

**Affiliations:** 1 The Social Institution, Research Department, Finland

## Background

The genetic background of the Finnish population has led to a unique epidemiology for some the rare hereditary disorders. In particular the incidence of PKU-is very low, less than 1:100000. Finland has screened only for congenital hypothyroidism from cord blood since 1984. A proposal to start a pilot study on screening with MS/MS with the cost estimation of 1-3€/newborn necessitated a Health technology assessment (HTA). In this article the major problems encountered during the assessment process and thereafter are presented.

## Material and methods

The original HTA project started form identifying the possible disorders to be screened for Finland, evaluating the possible costs of building up the screening organization, cost-effectiveness, organizational and ethical consequences [1,2]. The original documents from the HTA project and from the national screening committee at the ministry of social affairs and health were used.

## Results

Lacking reliable data on incidence, natural course of the possible disorders, sensitivity and specificity of the screening tests, effect of early diagnosis and early treatment raised many ethical questions. It became evident that a thorough ethical evaluation was needed to answer questions like “Can infants become the focus of research to answer the unknown? When is it acceptable to screen for many to find a few?”. Modeling a screening organization does not guarantee that it will work in real life but the estimated costs were 45€/newborn for finding 5-10 children with a rare metabolic disorder per year in Finland. The annual birth rate in Finland is approximately 56000.The national screening organization has discussed the topic at 17 meetings during 2003-2012, the chairpersons and committee members have changed. Seminars and workshops with content experts have been organized to answer the identified critical questions, yet more information was needed. A national pilot study was withdrawn; a local feasibility study was conducted. An HTA report on PKU screening when neither parent is of Finnish origin was conducted in 2008 [3].

## Conclusions

The original assumptions on costs were fictional. A continuous conversation with the screening committee and the content experts has been necessary and understanding the complexity and consequences of the decision increased during the years. The ongoing changes in structuring health and social care and the planned changes in legislation affect how national screening programs can be implemented. Screening for metabolic disorders in newborn has so far not yet been expanded.
